# Supportive or Confining? The Impact of War Metaphors From the COVID-19 Pandemic on Persons With Disabilities in Mainland China

**DOI:** 10.3389/fpubh.2021.720512

**Published:** 2021-09-20

**Authors:** Ren-Xing Chen, Zhong-Ming Ge, Shu-Ling Hu, Wei-Zhong Tang

**Affiliations:** ^1^Department of Social Security, School of Labor and Human Resources, Renmin University of China, Beijing, China; ^2^Department of Social Work, School of Philosophy and Social Development, Shandong University, Jinan, China; ^3^Weizhong Children's Rehabilitation Center, Jinan, China

**Keywords:** COVID-19, war metaphors, persons with disabilities, pragmatism, identity segment

## Abstract

Ensuring the well-being of persons with disabilities (PWDs) is a priority in the public sector during the coronavirus disease 2019 (COVID-19) pandemic. To contain this unprecedented public crisis in China, a set of nationwide anti-epidemic discourse systems centered on war metaphors has guided the epidemic's prevention and control. While the public is immersed in the joy brought by the stage victory, most ignore the situation of the disadvantaged PWDs. Accordingly, this study adopts and presents a qualitative research method to explore the impact of war metaphors on PWDs. The results showed that while there was some formal and informal support for PWDs during this period, they were increasingly marginalized. Owing to the lack of a disability lens and institutional exclusion, PWDs were placed on the margins of the epidemic prevention and control system like outsiders. Affected by pragmatism under war metaphors, PWDs are regarded as non-contributory or inefficient persons; therefore, they are not prioritized and are thus placed into a state of being voiceless and invisible. This research can provide inspiration for improving public services for PWDs in the context of COVID-19.

## Introduction

Coronavirus disease 2019 (COVID-19), which emerged at the end of 2019, spread rapidly worldwide because of its fast transmission speed, high infection rate, and difficulty to prevent and control. COVID-19 has become an urgent public health issue that threatens human life and health. Accordingly, the World Health Organization (WHO) designated COVID-19 as a global health emergency of international concern on January 30, 2020. With the recent large-scale spread of COVID-19 infections in India, there is a trend of resurgence. At the time of this writing, more than 180 countries have reported confirmed cases. The cumulative number of confirmed COVID-19 cases worldwide is 170,178,953, with 3,538,858 deaths (1). Notably, studies have shown that persons with disabilities (PWDs) are more likely to have underlying health problems and live in collective care settings, which increases the risk of infections and other secondary problems (2). It is estimated that there are more than 1 billion people—equivalent to about 15% of the world's population—who have some form of disability, and this number is increasing due to the aging of the population and the growth of chronic diseases (3). The WHO calls for disabilities to be regarded as an important public health issue and included in the work of the health sector. Therefore, in the context of a major public health event such as COVID-19, more priority should be given to PWDs, which are perceived as vulnerable and susceptible populations.

Compared with the general public, PWDs are at a relative disadvantage in terms of physical functioning, economic status, education, and information access, which leads them into the double jeopardy of marginalization regarding their preventive health care and nursing during the COVID-19 period (4). Since the outbreak of COVID-19, hospitals, clinics, and rehabilitation institutions have been considered as potential sites for virus transmission, and many of these facilities have reduced their activity or have been completely closed (5). Consequently, appropriate treatment and rehabilitation services are not available for PWDs during the COVID-19 outbreak. In addition, the experience of a lack of freedom caused by behavioral restrictions during home quarantine makes people with physical, intellectual, and mental disabilities more likely to develop serious mental health problems such as cabin fever (6). In these particular situations, PWDs are vulnerable to isolation and psychological distress, mainly because of social distancing and quarantine measures. Therefore, COVID-19 poses a greater threat to PWDs in terms of public health, society, and politics. As a key event in the life course of PWDs, COVID-19 will have an immeasurable impact on them. If necessary measures that truly meet the actual needs of PWDs are not taken, it will be detrimental in protecting them from COVID-19.

At the end of 2019, the Chinese people were preparing for the country's largest traditional festival, the Lunar New Year. Those who work in other cities would go home to spend the Spring Festival with their families, and this is also regarded as the world's largest population migration (7). This undoubtedly accelerated the speed and breadth of the spread of COVID-19 in China. Subsequently, China immediately closed off Wuhan, starting from January 23, 2020, and put the country into a state of emergency; moreover, it also activated the highest-level emergency response mechanism to control the spread of COVID-19. China then adopted a series of prevention and control measures, such as blocked cities and villages, and strictly restricted population movements and collective activities that may spread the virus. COVID-19 attracted the attention of domestic and foreign media and became a priority agenda in the process of the epidemic. Given this, studies have shown that war metaphors are pervasive in the media coverage, with epidemic control as a war that must be won and COVID-19 as the enemy (8). Although the use of the rhetorical method of war metaphors is more common in the world, this feature is particularly obvious in Chinese media.

Previous studies have explored the war metaphor during the COVID-19 outbreak and its impact on epidemic prevention and control. There are also numerous studies discussing the reasons for the success of China's epidemic prevention and control and the key influencing factors and main mechanisms. However, most existing studies have overlooked the marginalized groups of PWDs. What do the war metaphor and epidemic prevention and control measures dominated by this discourse mean to PWDs? What impact does it have on them? Few researchers have focused on this issue. Therefore, based on the qualitative research method, this study is an exploration of the impact of war metaphors—whether it is positive or negative—on PWDs.

## Literature Review

### War Metaphors and COVID-19

As a linguistic device, a metaphor is deeply rooted in language, thought, and action, pervading everywhere in everyday life. Accordingly, the “covert” and “possibly unconscious” intentions of language users can be revealed through critically analyzing metaphors (9). Lakoff and Johnson (10) point out that the essence of a metaphor is a common and unavoidable way of thinking. We often unconsciously adopt metaphorical systems to understand abstract things—to understand and experience another kind of thing with one kind of thing—claiming that metaphors exist not only in language but also in daily life, thoughts, and actions (10). Studies have found that war metaphors or military metaphors are important frameworks for the media to construct a disease. Such metaphors can create a sense of urgency, provide a common basis for thinking and action, and are an important means of motivating society and mobilizing people (11). Diseases are often presented in the form of war metaphors such as strikes, attacks, invasions, and spreads, which are common metaphors used to describe illness. The coping methods used by humans to fight diseases include defense, struggle, and resistance (12).

The use of war metaphors can be traced back to Pasteur and Koch's early research on infectious diseases. The most classic study comes from Sontag's (13) research of the metaphor of disease and acquired immunodeficiency syndrome (AIDS). She revealed the process of the metaphorical construction of the disease. This research shows that what people see is not the real appearance of disease but is rooted in the special cultural context and social background of each period. As Sontag clarified, the metaphor of “disease is war” is dominant in people's conceptualization of disease. In the public health discourse system guided by the war metaphor, disease is often described as an enemy invading society, and efforts to control the spread of a pandemic and reduce infection and mortality are referred to as “a fight, a struggle or a war” (13).

The image system of war metaphors helps to provide us with a view of COVID-19 in some aspects. Through the use of war metaphors, the disease itself is endowed with social significance in addition to its biological meaning (11). To provide the audience with an idealized vision of society, the selection or presentation of metaphors by news media is conscious and often carries the purpose of persuasion. The “unverifiable” nature of metaphor and its selective “reinforcement” and “concealment” toward reality contribute to the expression of the news media's political stance (9). Notably, the power of war metaphors is that they can make people in a fearful situation take defensive actions and also mobilize people to cope with emergencies (12).

The war metaphor is consistent with the current context of the prevention and control of COVID-19. As the most authoritative form of media in China, the war metaphor is the dominant framework for the *People's Daily* to report on COVID-19. It is described as the enemy of the whole nation, emphasizing the ruthless and barbaric characteristics of the virus such as the ferocious virus, cunning human natural enemies, etc., thus classifying the virus as an evil other, highlighting its antagonistic relationship with humankind (14). The metaphor, envisaging dreaded diseases “as an alien ‘other,' as enemies” in modern war (13), attempts to create a sense of urgency and mobilize the public to fight against a common threat.

### COVID-19 and Persons With Disabilities

The COVID-19 outbreak has created continuous challenges for PWDs. As they face many disadvantages in health care services and community life, coupled with physical defects and a series of social barriers, the cumulative effect of disadvantage puts them at greater risk during the COVID-19 pandemic (15). They had to interrupt their routine activities, were forced to suspend their work, and could not participate in community life and enjoy some services. In particular, PWDs living in collective care settings, such as group homes, centralized nursing institutions, and long-term residential care facilities, are at greater risk of infection and death—which are several times higher than the general population (16). In addition, the impact of COVID-19 on PWDs shows certain differences among the different types of PWDs. Among them, those with intellectual and developmental disabilities (IDD) are more susceptible to the impact of COVID-19, which occurs in the areas of their physical and mental health, the social sphere, and setbacks to human rights (17). Related data analysis results show that the death rate of those with IDD is two to six times higher than that of people without IDD when at risk of COVID-19 infection, and they are more likely to die from pulmonary complications (2).

To achieve normal social participation and social integration, PWDs often have higher needs than non-disabled people in terms of education, employment, healthcare services, and media support (15). However, services related to PWDs were forced to cease owing to the impact of COVID-19 and the implementation of a strict lockdown policy during the pandemic. The allocation of medical products and related services poses a significant threat to the human rights of PWDs, and certain states in the United States have even formulated disability-based exclusion from lifesaving treatments (4). Specifically, telemedicine is seen as a way to partially compensate for the health care needs of PWDs during home quarantine and reduce the risk of COVID-19 infection and save costs. However, when using telemedicine, PWDs still face many barriers, primarily including infrastructure and access barriers, operational challenges, regulatory barriers, communication barriers, and legislative barriers (18).

Even more serious is that COVID-19 poses significant challenges to the rehabilitation services, care, and mental health of children with disabilities. Owing to the lack of an inclusive humanitarian response, neglect, abuse, and separation from family members may put disabled children in desperate circumstances and cause lifelong trauma (19). Therefore, to support PWDs in responding to the public health crisis, relevant suggestions should be proposed. For example, this could be data collection about disease, recovery, and mortality rates from COVID-19 among PWDs. Moreover, short-term and long-term compensation measures and related plans, such as medical treatment, rehabilitation, and vaccination, could be developed (4).

In summary, existing studies have focused not only on the discourse system of war metaphors related to COVID-19 but also on the difficulties and challenges faced by PWDs in this major crisis. Accordingly, scholars have put forward corresponding countermeasures and suggestions from the perspectives of the government, social organizations, and public health. However, the critical impact of war metaphors on PWDs during the COVID-19 outbreak has not been systematically examined in the existing literature. Therefore, based on the qualitative method, we attempt to explore this important topic from the subjective perspective of PWDs to bridge the gap in current research.

## Theoretical Perspective and Methods

### Theoretical Perspective: War Metaphors During the COVID-19 Outbreak in China

Based on an analysis of existing literature and news reports, this paper summarizes the manifestations and specific features of war metaphors during the prevention and control of COVID-19 in China. This mainly includes two aspects: first, the conceptual set of war metaphors in China; second, the top-down command chain and organization-action system under the war metaphor.

#### The Conceptual Set of War Metaphors in China

The war metaphor in medical discourse is used to connect the two conceptual systems of medical treatment and war—the mapping relationship between the source and target domains or the tenor and vehicle (20). Thus, it constitutes the conceptual metaphor of “medical treatment is war”, which is coherent with the metaphor. Whether it is an official's speech or a news report about COVID-19, war metaphors are used as the dominant narrative method during the anti-epidemic period, which has penetrated the entire society. This is related to the cultural heritage of the communist armed struggle during the Chinese Revolution (21). In addition, the war metaphor is the dominant framework for the *People's Daily*—which is regarded as the most authoritative medium in China—to report on COVID-19. Moreover, this “epidemic prevention and control war” is divided into “technical assault war”, “scientific research war”, and “material support war”, and it is still a “general war” and a “people's war” (14). Frequently used terms include “war of containment” (*zujizhan*), “all-out war”, etc. War metaphors convey a strong sense of urgency and can also be used as emotional mobilizations to call on citizens to act in compliance with mandatory measures for epidemic prevention and control (8).

**Figure 1:**
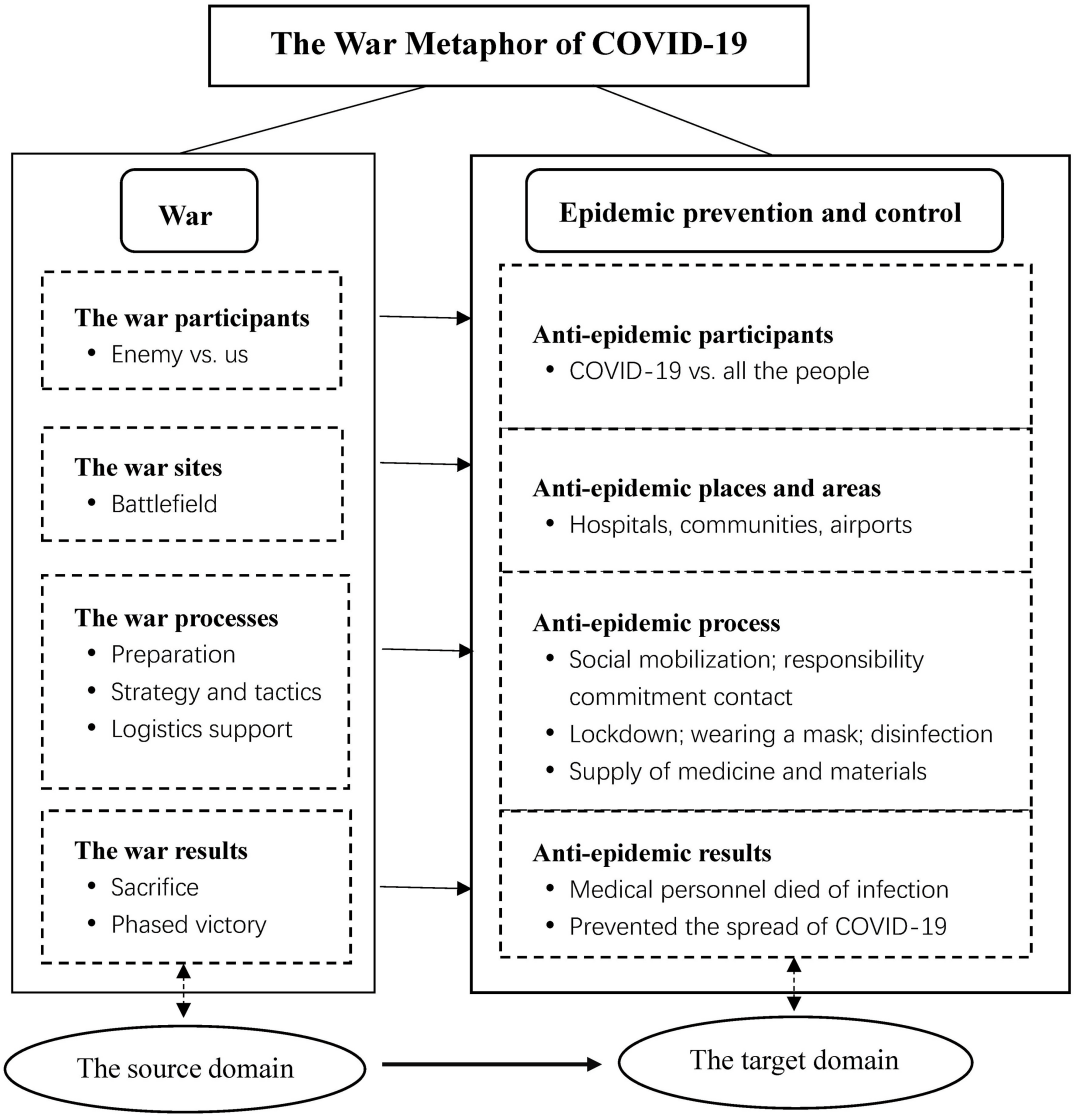
The mapping relationship between the war metaphor and COVID-19.

To more clearly present the war metaphor during the COVID-19 period, we enumerated the mapping relationship between war (source domain) and anti-epidemic (target domain) (as shown in Figure 1). The elements presented in Figure 1 include the following: (1) The war participants, including the enemy (COVID-19) and our side (medical staff represented by Zhong Nanshan; frontline epidemic prevention personnel in the community; all the people, etc.). (2) The war sites—the key places and areas for epidemic prevention and control, such as hospitals, communities, and airports. (3) The war process, including pre-war preparations (e.g., social mobilization, epidemic prevention training, and officials signing responsibility commitment contracts); formulating strategies and tactics (lockdowns of cities and villages; wearing masks, disinfection and sterilization, resource allocation, personnel transfer, etc.); logistics support (the supply of drugs and medical devices, and the support of living materials). (4) The war results, such as sacrifice (medical staff who died due to infection), phased victory (preventing the spread of the epidemic), and triumphant return (assisting medical staff in the hardest-hit areas to return home).

Based on the mapping relationship between the source and target domains, we can clearly see how the war metaphor is constructed in China. Affected by the war metaphor, China launched a nationwide campaign against COVID-19 and has managed to contain this unprecedented public health crisis. In particular, this is due to the relatively hardcore epidemic prevention and control measures under the war metaphor (22). Therefore, comparing COVID-19 to war will increase the public's sense of urgency and crisis and serve as a significant social mobilizing force for the whole country to unite in response to this unprecedented public crisis.

#### Top-Down Command Chain and Organization-Action System

The war metaphor is not only a mapping relationship between war and COVID-19 but also a mapping of war practices and experiences into the specific practices of epidemic prevention and control. Such a metaphor constructs a top-down national anti-epidemic strategy system and shapes the image of national unity to win the fight against COVID-19 (8). To address this unprecedented public health crisis involving COVID-19, an effective chain of command should be established to confront this situation, which is essential for effective communication and interdepartmental coordination.

On January 20, 2020, the Joint Prevention and Control Mechanism of the State Council was officially launched. On January 25, 2020, China established the Central Leading Group for COVID-19 Prevention and Control (CLG) as the highest decision-making institution and command center for China's response to COVID-19. The instruction of the CLG is considered an uncompromising political task that all participants must complete. Simultaneously, governments at all levels have also established epidemic prevention and control centers, thus forming a top-down command chain. Notably, war metaphors have certain advantages in mobilizing resources to respond to crises. The state will uniformly allocate resources and deploy human and material resources in a top-down manner so that rapid social mobilization can be conducted (23).

To win the war against COVID-19, China has formed a rigorous top-down organization-action system. First, strict anti-epidemic strategies and measures have been formulated, and a nationwide lockdown and home quarantine plan have been urgently implemented to restrict the movement of non-essential people. Second, China provides political incentives for local officials with outstanding contributions through “battlefield promotion”, thereby prompting them to perform better in the anti-epidemic war (22). By allowing civil servants to sign a “responsibility contract”, which is similar to a “military order”, they are required to perform their duties. Various disciplinary actions, including suspension, demotion, and dismissal, have been taken against cadres for their poor performance in the fight against COVID-19. For example, to support Wuhan, the hardest-hit area, China has selected medical personnel from various provinces and cities. Moreover, in this regard, they have taken an oath before going to the front line of the epidemic, which is seen as an effective form of mobilization.

The war metaphor is the dominant framework for constructing COVID-19 in the Chinese media. It emphasizes national speed and power, enhances the collective identity and national identity of the audience, and realizes the authority and legitimacy of anti-epidemic (14). The war metaphor regards COVID-19 as an “enemy” that invades the country, and every citizen's body may become an occupied “city”. Each aspect of the cognition, response, and action involving COVID-19 is expressed in a war-like expression, aiming to create an atmosphere of urgency and crisis, and mobilizes the population to stand on their own posts to win the anti-epidemic war.

### Methods

#### Research Design and Sampling

To explore the impact of the war metaphor under COVID-19 on PWDs, a qualitative research method was chosen as the design guide for this study, using the interpretivist approach to understand the interviewees' understanding of the world of social life. It allows researchers to be deeply involved in the observation and understanding of the participants with a natural attitude and develop a familiar relationship with them, and the resulting observations and interactions led to genuine revelations (24). Moreover, it can critically reflect on the participants' cultural interpretations of their social situations as well as the themes and assumptions they bring to the study (25).

We recruited the interviewees through online methods. We posted recruitment advertisements on WeChat, QQ, and Weibo, the three largest online platforms in China. The advertisements explained the research content, research objectives, and basic requirements of the interviewees. After the registration deadline, we received 25 letters from potential participants. In order to collect as much potential information as possible related to the research topic, this study adopts purposive sampling to select the interviewees who can provide the most information (26). Our main criterion for selecting interviewees is “the richest information” in order to achieve “saturation of the most diverse information” (26). After the researcher's preliminary trial interviews and screening, 11 interviewees were finally determined. The age of the interviewees ranged from 16 to 48; the group comprised six males and five females, covering different categories and degrees of PWDs. Geographically, they came from the southeast coastal areas (five), the central-western region (four), and the northeast region (two). When selecting the participants, we tried to consider factors such as age, gender, household registration, and education. Other important criteria were related to the type of disability and degree of disability, as they provide a different subjective experience. This study enhanced the reliability and validity of the research by improving the heterogeneity of the interviewees. In addition, to explore the research issues from a multidimensional perspective, through purposive sampling, we selected three staff members working in the Disabled Persons' Federation and three members of the community serving PWDs to achieve a more comprehensive and detailed understanding. The descriptions of the research participants and their general characteristics are presented in Tables 1, 2.

Table 1Description of research participants.

**Table:** 

**1. Participant I: Persons with disabilities (PWDs)**							
**No**.	**Gender**	**Age**	**Household registration**	**Education**	**Disability type**	**Degree of disability**	**Current status**
C-1	Female	32	Urban, Beijing	University	Physical	Moderately disabled	Company's cashier
C-2	Male	28	Urban, Shenzhen	Senior in high school	Physical	Profoundly disabled	Phone customer support
C-3	Male	48	Rural, Changchun	Primary school	Visual	Profoundly disabled	Unemployed single man
C-4	Male	45	Rural, Jinan	Senior in high school	Visual	Profoundly disabled	Blind masseur
C-5	Female	26	Urban, Jinhua	Post-graduate	Cerebral palsy	Moderately disabled	Company staff
C-6	Female	36	Rural, Wuhan	Primary school	Mental	Mildly disabled	Unemployed
C-7	Male	16	Urban, Guizhou	Primary school	Autism	Moderately disabled	Stay at home
C-8	Female	33	Rural, Zhengzhou	Vocational	Intellectual	Mildly disabled	Pastry chef
C-9	Male	36	Urban, Chengdu	University	Hearing	Profoundly disabled	Illustrator
C-10	Male	37	Rural, Shanghai	University	Physical	Moderately disabled	Civil servant
C-11	Female	42	Rural, Jinan	Primary school	Intellectual	Mildly disabled	Cleaning crew

**Table:** 

**2. Participant II: Faculty of department of disabled persons' federation**				
**No**.	**Gender**	**Age**	**Education**	**Job title**
F-1	F	34	University	Deputy Chief of Rehabilitation Department
F-2	M	42	Senior in high school	Staff of Disabled Persons' Federation
F-3	F	25	University	Staff of Disabled Persons' Federation

**Table:** 

**3. Participant III: Staff in the community who provide services for PWDs**					
	**Gender**	**Age**	**Education**	**Job title**	**Work experience**
S-1	M	38	University	Staff in X district	12 years
S-2	F	36	Senior in high school	Staff in J district	8 years
S-3	F	28	Senior in high school	Staff in N district	4 years

**Table 2:** General characteristics of the research participants (*N* = 11).

**Variables**		***N* (%)**
Gender	Male	54.5
	Female	45.5
Age	≤30	27.3
	30–40	45.5
	40–50	27.3
Household registration	Rural	55.5
	Urban	45.5
Education	Primary school	36.4
	Senior in high school	27.3
	University and above	36.4
Disability type	Physical disability	27.3
	Visual disability	18.2
	Intellectual and developmental disability	9.1
	Hearing disability	36.3
	Mental disability	9.1
Degree of disability	Mildly disabled	27.3
	Moderately disabled	36.4
	Profoundly disabled	36.4

#### Data Collection and Data Analysis

Semi-structured in-depth interviews were conducted to collect raw data. Based on the research questions and content, we created an interview outline. Subsequently, we revised the interview outline based on the trial interviews. For PWDs, the content of the interview involved how they viewed the war metaphor under COVID-19 and what this metaphor meant to them. For the Disabled Persons' Federation and community workers, the questions focused on their experience of providing support, benefits, and services for PWDs. These included their methods, service descriptions, and their thoughts and opinions on PWDs during COVID-19. We used WeChat voice or telephone interviews, and each participant's interview consisted of two rounds. The first round followed the interview outline; subsequently, the interview contents were sorted promptly to find out the missing contents or the contents that needed another interview. The second round of interviews were then conducted, and each one was audio-recorded with the consent of the interviewees and lasted between 60 and 90 min. Finally, the recordings were transcribed by authors.

We adopted an inductive thematic analytic approach to categorize and analyze the raw data; accordingly, the NVivo 10 software package was used as an analysis tool. Following the guidelines laid out by Braun and Clark (27) in a recursive process, the data encoding and thematic analysis mainly included three steps. The first step was open coding based on thorough reading and a comprehensive understanding of the interview data to prepare the primary coding list and make important notes and memos. The second step involved the process of categorization, in which the primary coding with similar content and logic was classified under the same category to form a secondary coding system. Finally, the third step was the process of abstraction and conceptualization, extracting the dominant core categories. When there was no new coding, we believed that we reached theoretical saturation; therefore, we stopped the further recruitment of interviewees.

We conducted a dialog with the existing literature to examine the characteristics and manifestations of war metaphors during COVID-19. We analyzed government-promulgated policy documents and files related to PWDs during the COVID-19 epidemic and improved the validity of the research results by supplementing evidence from multiple sources. With reference to the framework proposed by Lincoln and Guba (28), this study mainly conducts reliability and validity analysis from four dimensions. (1) Credibility, that is the truthfulness of the qualitative data. Researchers use interview techniques effectively to seek the authenticity of the data by different questioning methods, and conduct multiple checks on the interview data, pursuing the authenticity of it. (2) Transferability. Researchers made a thick description of the interviewee's experience, and fully presented the feelings, experiences and actions expressed by the interviewee. (3) Dependability. Interviews and data transliteration were done by the researchers in cooperation to ensure the stability and consistency of the data. (4) Confirmability. When analyzing and interpreting data, the researcher eliminates personal prejudice and remains neutral to ensure that the experience and facts expressed by the participants are truly presented. The ethics committee of the researcher's University approved the ethical issues involved in this study. Before the interviews and recordings, the researchers obtained written consent from the interviewees. To protect the privacy of interviewees, each participant was anonymized in this paper.

## Findings

**Figure 2:**
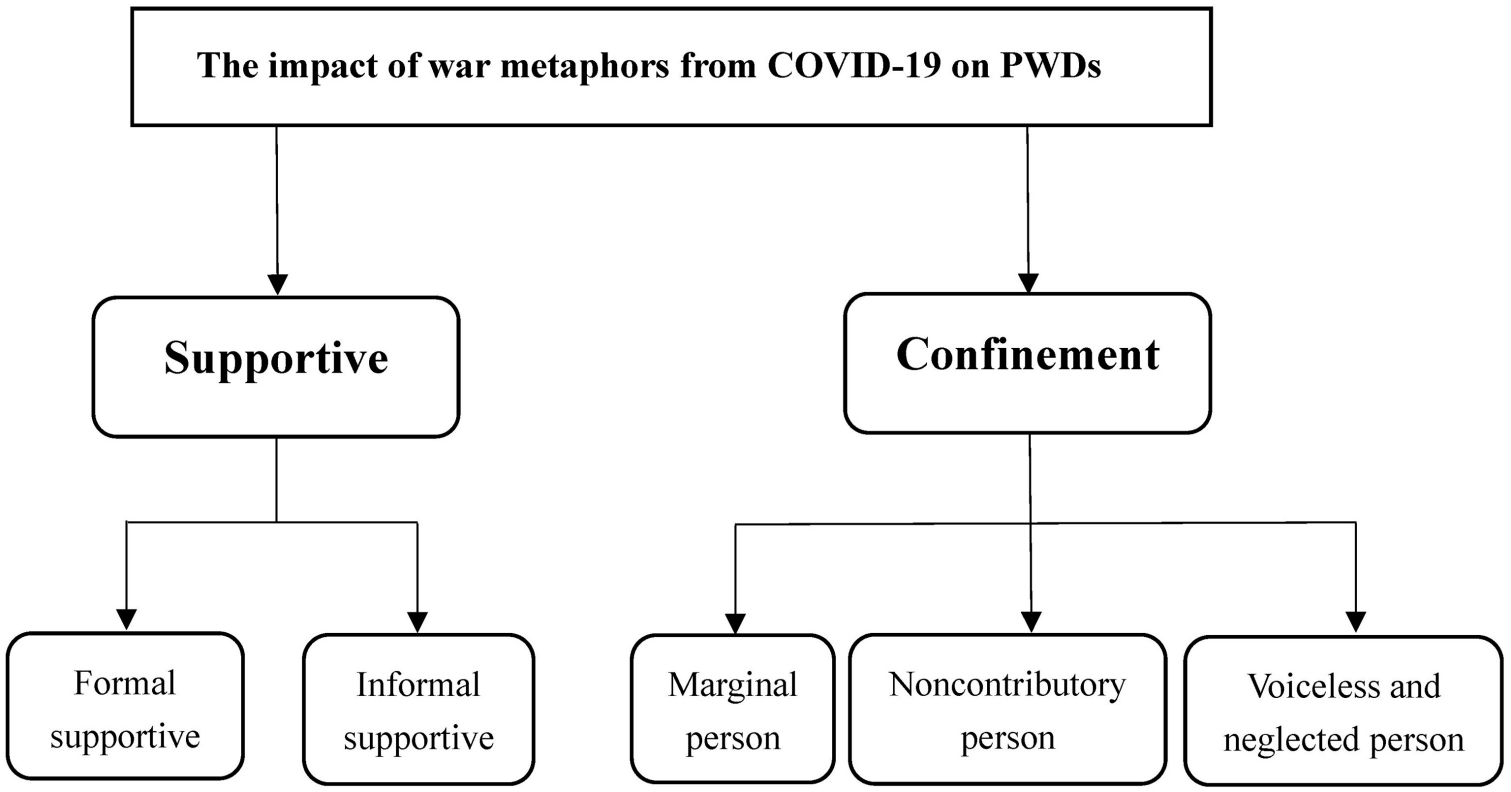
The logical framework of this study.

In the preceding article, this study focused on the manifestations and specific features of war metaphors during the prevention and control of COVID-19 in China. By introducing the measures and results of China's anti-epidemic efforts, we can gain insight into the construction logic of war metaphors and lay a foundation for understanding the situation of the PWDs in China. In the epidemic prevention and control system dominated by war metaphors, although the PWDs have received formal support from the government and informal support from civil society, on the whole, the PWDs are increasingly marginalized. Affected by the war metaphors, the PWDs are regarded as noncontributory person and therefore are not given priority. This has led to the allocation of public health resources to focus more on anti-epidemic work rather than the PWDs care, which made the PWDs suffer a loss of medical resources and a higher risk of life. We established the logical framework of this study based on the interview data (Figure 2).

### Sounds Like Positive Protection: Minor Increase in the Well-Being of PWDs Under War Metaphors

PWDs are considered to be at greater risk in this public health crisis; therefore, healthcare and related services should be given priority. The Convention on the Rights of Persons with Disabilities (CRPD) states that all measures necessary to ensure the protection and safety of PWDs are provided to guarantee the right to life and health. Affected by the war metaphor, the government formulated relevant welfare policies to deal with this crisis and launched a wide range of social mobilizations to solve the difficult situation of PWDs. We divided these measures into formal support from the government and informal support from civil society.

#### Formal Support From the Government

First, necessary measures have been taken to guarantee the basic living standards of PWDs. A typical feature of war metaphors is the establishment of a top-down command chain and a rigorous organization-action system. Notably, to alleviate the impact of COVID-19 on the daily life of PWDs, the government has rapidly increased the living allowance for the poor PWDs and the nursing allowance for the profoundly disabled (two-subsidies), which is regarded as the core of social welfare for PWDs in China. Therefore, using Wuhan as an example, during the epidemic, the government increased the living allowance by 50 yuan from the original standard of 130 yuan per month (urban areas)/110 yuan per month (rural areas). Further, the nursing allowance was increased from 100 yuan to 150 yuan per month. The government has appropriately relaxed the scope of social assistance—including more PWDs with difficulties—into the minimum living security system and temporary assistance system, and correspondingly raised assistance standards. In addition, the government has provided rehabilitation services subsidies and temporary price subsidies for PWDs, subsidies for home-assisted services for the profoundly disabled without work, and subsidies for nursing care for elderly PWDs. Based on this, a comprehensive guarantee system with Chinese characteristics was established.

Second, employment subsidies and employment services were required, which stabilized the employment rate of PWDs. COVID-19 has had a greater impact on the employment of the PWDs. Affected by the war metaphors, the state prefers the allocation of resources to the employment and income growth for the PWDs to ensure social stability and epidemic prevention and control. The government adopts preferential policies such as reduction or exemption of social insurance premiums, deferred payment of taxes and fees, and reduction or exemption of rents to support businesses or shops (blind massage institutions, etc.) run by PWDs to maintain normal operations. The government also provides incentives for companies to hire PWDs or reduce the risk of being laid off by offering job stability and recruitment subsidies. The government has increased employment opportunities for PWDs through the establishment of public welfare positions, subsidies for job hunting and entrepreneurship, and subsidies for job training. Methods such as centralized and decentralized, online and offline, and home delivery of courses are used to conduct vocational skills training for PWDs to enhance their employability. Moreover, the government provides employment assistance services for registered unemployed PWDs and provides unemployment insurance and unemployment subsidies to ensure their basic livelihoods.

Third, services for PWDs have been restored and promoted in an orderly manner, which has improved their social well-being. China's Disabled Persons' Federation promulgated the “Notice on Focusing on Special Operations for Visiting Families of Disabled with Special Difficulties under the Impact of the Epidemic”, emphasizing the development of visits and condolences. Community workers should visit families of disabled people who have special difficulties in basic daily care, medical treatment, and schooling due to the epidemic and coordinate efforts to solve practical difficulties. Communities conduct a guarantee contact system that coordinates Party members, cadres, community workers, and volunteers in communities (villages) and implement the insured contacts one by one for PWDs who are lonely or unable to take care of themselves for various reasons. Specific services include regular visits, policy publicity, voluntary services, agency assistance, and spiritual comfort to ensure that PWDs can receive timely and effective assistance when they encounter emergencies. In addition, the government has adopted corresponding incentive measures to encourage family doctors to provide services such as family beds and outpatient visits for PWDs on the premise of self-protection to ensure continuous and uninterrupted home services.

#### Informal Support From Civil Society

During epidemics, certain types of PWDs are unable to access communication and information platforms to acquire valuable information, which puts them in a more vulnerable position where they are more susceptible to virus attacks and impacts. This can be attributed to social, financial, and technological reasons (29). Affected by social mobilization in the context of war metaphors, relevant social organizations were encouraged to provide accurate and accessible health information about COVID-19 and methods of continuous self-protection for PWDs to compensate for the absence of government in this field. For example, Shouyuzhe, a non-profit organization in Wuhan that specializes in serving hearing disabilities, continuously updates the latest information about the epidemic by making sign language videos, which are seen as key to protecting PWDs during the epidemic.

Under the war metaphor, caring and non-disabled people support PWDs through various channels, which is regarded as a virtue and a commendable charity. Consequently, during the COVID-19 pandemic, there was a scattered patchwork of social support for PWDs. For example, the Beijing Disabled Persons' Federation has set up a 12385 psychological counseling service hotline to provide timely psychological support, emotional counseling, crisis intervention, and other mental health services for PWDs and their relatives to prevent extreme events caused by psychological stress. Some medical institutions provide online rehabilitation consultation and telemedicine rehabilitation guidance for PWDs to perform well in home rehabilitation training and epidemic prevention. In addition, some commercial organizations participate in helping PWDs improve their social reputation. For example, the Huaxia Insurance Company specially designed a COVID-19 exclusive insurance of 200,000 yuan for PWDs, and eligible PWDs can participate in the insurance free of charge.

### Actual Confinement: Further Marginalization of PWDs Under the War Metaphor

#### Reinforced Isolation: Further Social Distance From the Outside World

##### Outsiders: PWDs Were Placed on the Margins of the Anti-epidemic System

While COVID-19 is a fatal threat to all, the challenge is even more complex for PWDs, who have faced various forms of social exclusion—visible and invisible—during the pandemic. Strict home quarantine measures make it difficult for PWDs to interact with the outside world, which significantly increases their risk of being neglected or abused. Information accessibility is another challenge for PWDs, as much of the information is not converted into an accessible format (7). Consequently, PWDs cannot obtain accessible and valuable information, and the chains of their interactions with others are severed. This deepens the isolation and distance between PWDs and the outside world, making it more difficult for them to achieve social integration. In addition, during the pandemic, China developed an app called *Jiankangbao* that requires the individual to input their temperature, travel declaration, and scan code registration, which is regarded as a “passport” for citizens. However, this is a significant challenge for PWDs, especially those with IDD. To avoid causing trouble for others, most PWDs choose to stay at home instead of going out.

*In the early days of the outbreak, I had no idea what was going on and could only get bits and pieces of information from my family. That fear of the unknown has always affected me, making me feel like it's the end of the world*. *I tried to catch something, but it was a needless struggle. Suddenly, I feel that everything happening around me is so strange, I am like an invisible person, and the world seems to have nothing to do with me. (C-3)*

In addition, there are paradoxes and contradictions between strict epidemic prevention and control measures and the survival needs of PWDs under the war metaphor. To cut off the spread of COVID-19 through person-to-person transmission, social distancing is required to avoid direct contact with others. However, for certain categories of PWDs, social alienation and self-quarantine can be challenging (30). For example, for persons with hearing disabilities, oral language is critical to their communication, but the veil of a mask significantly limits their ability to communicate. Moreover, people with visual disabilities need to walk with the arms of others or get information by touching Braille characters along with the surfaces in public spaces. Some profoundly disabled people who cannot take care of themselves often rely on the care of others. Finally, it is difficult for those with IDD to understand the sudden home quarantine and related measures, which makes them extremely confused. Therefore, one sentence often mentioned by interviewees is “I seem to be an outsider”.

*I haven't been out for a long time, and it hasn't been communicated with my peers for a while (hearing disability) because wearing masks makes it difficult for us to read lip language. Further, seeing the entire face is very important for our communication. However, this was not possible. So, for me, I feel very lonely now. (C-9)*

##### Institutional Exclusion: Deepening Social Inequality

Institutional exclusion refers to the process of PWDs being excluded from the system and unable to obtain the support of necessary social resources because of the limitations or deficiencies of the system—thus becoming a more vulnerable group (31). This separation seems to be due to the lack of ways for PWDs to express their interests. However, essentially, the advantaged groups repel disadvantaged groups to protect their interests from losses in the process of resource allocation. It is perceived as a rationalized social deprivation caused by non-disabled people based on the negative perception of disability. This exclusion is reflected in the fact that experts in epidemic prevention and control, led by medical professionals, often associate disability with adverse health conditions. This bias has a decisive influence on medical futility decisions (32). This prejudice against disability prevents PWDs from obtaining the equipment or medical services they need; it may also lead to them not being prioritized in resource allocation. Therefore, it can be said that the outbreak of COVID-19 is questioning the commitment to equality.

The consequence of institutional exclusion is social inequality led by the concept of quality of life, which holds that PWDs have a poor quality of life and a lower life expectancy and even account for a waste of resources. Experts who controlled the discourse will evaluate them through wartime triage so that those with the greatest chance of survival and successful treatment will be given priority regarding medical and healthcare services (33). In this situation, priority will be given to young and healthy groups, rather than disadvantaged and marginalized groups such as PWDs. Therefore, PWDs are excluded from priority access to health services and related anti-epidemic medical resources, even if they are in greater need of these services than non-disabled individuals.

The larger coordinates of the threat became apparent that pre-established frameworks for ethical decision-making in health crises excluded PWDs because of preconceived notions about their quality of life (34). It can be concluded that judgments about their quality of life are typically made by someone with no experience of disability as well as from an ableist perspective (35).

*I need to take medication (for mental disability) regularly; otherwise, it will get worse. However, I did not prepare enough pills. Strict lockdown was adopted during the epidemic, and I could not go to the pharmacy or hospital to buy medicine. I contacted the community director, and he told me, “At present, it's a wartime situation, all resources are concentrated on the front lines, and I really can't take care of you”. In their opinion, the disabled seem to be unimportant, and mental health is far less important than physical pain. (C-6)*

#### Non-contributory Person: the “Product” of Pragmatism Under the War Metaphor

##### Perception Biases Under War Metaphors: PWDs Are Considered Non-contributory

As suggested in traditional Chinese culture, PWDs are perceived as individual tragedies, their impaired body implies a moral or social loss, and they cannot fulfill their responsibility to their family and society (36). In Chinese history, PWDs are called *canfei*, which means useless and incapable, and they were given negative labels such as “parasite”, “crap”, “dependency”, “burden”, and “troublesome” (24). However, the requirements for individuals under war metaphors are highly efficient. They must be “high-quality”, “quality-guaranteed”, “easy to use”, “usable”, and “capable of winning”. Obviously, PWDs are regarded as unqualified and inefficient “parts” of the giant political-economic machine under the war metaphor (24) because they are considered to be unable to contribute to the victory of this anti-epidemic war and thus become the “invisible person”.

Under the war metaphor, to win this anti-epidemic war, those who were effective and non-disabled were mobilized, such as medical workers and scientific researchers. As they are regarded as warriors who can win the war, these people should rush to the front line for the greatest possible guarantee of victory in the war against COVID-19. The war metaphor also encourages “pioneers” and “warriors”, such as medical staff and community volunteers who are on the front lines of the anti-epidemic war. They have been given the characteristics of “indomitable fighters”, “daring to fight hard battles”, and “brave and fearless”. The title of “anti-epidemic hero” emphasizes their contribution to the prevention and control of the epidemic. However, affected by the war metaphor, we hardly see the participation of PWDs in the process of epidemic prevention and control. As PWDs are labeled as useless and noncontributory, they are regarded as a burden and a hindrance to epidemic control. In this context, non-disabled people expect PWDs to stay at home and not come out to cause trouble to others and society.

*In the critical period of epidemic prevention and control, I applied for the community pioneer post to participate in the fight against COVID-19, helping to do some basic work such as distribution of supplies, epidemic publicity, and temperature measurement. However, the community director rejected my application and told me*, “*You can take care of yourself during the home quarantine period, you're not qualified for these works, please don't cause us trouble”. (C-1)*

##### Pragmatism Under War Metaphors: Personal Sacrifice Seen as a Virtue

In response to this unprecedented public health crisis, China immediately established a top-down militarized epidemic prevention and control system, with resource allocation under the unified command of the state. The war metaphor reinforces the concept of “a community of common destiny”, brings all people into the united front, and requires them to obey the requirements of epidemic prevention and control. Therefore, sacrificing part of one's personal interests is regarded as a virtue because it is conducive to forming an atmosphere of unity to fight COVID-19. Collectivist values strengthened by communist ideologies inherently require citizens to exercise self-restraint when individual interests clash with collective interests and also require individual interests to be subordinated to collective interests (22). Certain forms of personal sacrifice are still considered a virtue in contemporary China, despite the powerful promotion of individualist values in society through marketization (37).

*At present, our country is in distress, and all people should unite to fight against COVID-19. Residents in the community must strictly abide by relevant rules. What does personal gain or loss matter in the face of national security? Although there is little that PWDs can do, they can at least do will in home quarantine. (S-1)*

Pragmatism, derived from war metaphors, has been the guiding principle in the entire epidemic prevention and control process. Pragmatism refers to the idea of preserving the basics and the overall situation—it is possible to give up the interests of the minority to protect the interests of the majority. Therefore, the resource allocation method dominated by pragmatism follows distributive rather than formal justice. Distributive justice refers to the fair allocation of resources and services in healthcare. This is rooted in the principle of maximizing benefits for the largest number of people (38). As mentioned above, PWDs are regarded as useless, inefficient, and unable to make contributions. Consequently, the interests of PWDs as a minority are shielded during the anti-epidemic war. In this context, the actions of PWDs seeking personal interests are seen as selfish and disregarding in the context of the overall situation.

*In fact, we have also noticed the difficulties and challenges faced by the disabled. But we can't help it. We only have three staff members in our community and a few volunteers, and there is a shortage of workforce. The current focus is on the frontline of the fight against the epidemic, with no regard for the disabled. In addition, most of them stay at home with their families to take care of them. We mainly focus on those who are in and out of the community. (S-3)*

#### Lack of a Disability Lens: PWDs Are Voiceless and Neglected

##### One-Size-Fits-All: The Product of the Dominating Ableist Perspective

Affected by the war metaphor, PWDs are regarded as marginalized groups that will not be prioritized. This kind of discourse often immerses us in a society dominated by the ableist perspective and neglects the needs of PWDs (7). Moreover, almost all decisions and plans are made by experts in a superior position, with no participation of the disabled. This has led to the formulation and implementation of one-size-fits-all epidemic prevention and control measures without considering the special difficulties and challenges faced by PWDs. This means that PWDs will receive the same treatment as non-disabled people in the event of a pandemic. This one-size-fits-all standard and lack of disability lenses have led to greater inequity. In some cases, ignoring the rights of PWDs can be fatal (7).

*During the epidemic, I did not receive any special care or support. I have expressed my difficulties and needs to the Disabled Persons' Federation, but they did not respond. I made suggestions to them more than once, hoping they would consider PWDs when formulating relevant policies, but none of these suggestions worked. (C-11)*

This neglect of PWDs and the one-size-fits-all approach is reflected in relevant legal documents and policy texts. For example, the Law on Prevention and Treatment of Infectious Diseases (LPTID) does not mention the need for PWDs in an emergency public health crisis (COVID-19). The notice on social assistance during COVID-19 prevention and control did not mention PWDs or list them as the target of special assistance. In addition, the Ministry of Civil Affairs has successively issued more than 10 documents on social assistance for the extremely poor groups, which stipulates that the recipients of assistance are minimum living-hood guarantee (MLG) recipients, special hardship people, etc. However, there is no mention of the disabled. Further, the neglect of PWDs is also reflected in the lack of statistics related to them during the COVID-19 pandemic, which makes it difficult to accurately grasp the impact of the epidemic on them as a whole and the related trends.

*Although the law clearly requires equal treatment of PWDs, in fact, no one except family members cares about us sincerely. PWDs often remain an afterthought, living as invisible citizens without any sense of existence. (C-8)*

##### Emphasis on Unity Over Individuality: The Individualized Differences of PWDs Are Ignored

The epidemic prevention and control measures under the war metaphor emphasize coordinating all the activities of the nation as a whole—focusing on the unity of the majority rather than the individual differences of the minority. To win this anti-epidemic war, most measures tend to adopt standardized and unified methods because this is cost-saving and efficient. Owing to the lack of personalized prevention policies for the differences in PWDs, they are left behind. For people with hearing and speech disabilities, most news coverage of COVID-19 is without sign language, which increases the barriers for them to obtain valuable information. The lack of publication of Braille information on COVID-19 also made it difficult for the visually disabled to access relevant information on time. During the home quarantine period, rehabilitation services for children with disabilities were suspended. Therefore, interviewees often refer to themselves as “the invisible person who has been forgotten in the corner”.

*During the epidemic, almost all institutions and services were suspended, and I was isolated from the outside world. At that time, the responsibility of taking care of me was entirely borne by my family, and there was almost no support from others. I felt very depressed and could not do anything, like a burden on my family and society. I was in a state of depression, anxiety, and hopelessness at that time. (C-7)*

##### Identity Segment: Eligibility for Benefits Based on Poverty Rather Than Disability

We reviewed policy documents on social welfare and social assistance issued during the COVID-19 pandemic. Based on an analysis of the policy text, we found that eligibility for such assistance was based on whether the applicant was in poverty, which was measured by economic income, and whether they were supported by family members rather than disability identity. For example, we extracted the definition of welfare recipients in the “Notice on Protecting People in Difficulties During the Period of Epidemic Prevention and Control”: “During the COVID-19 pandemic, temporary assistance will be given monthly to those in need, such as those living with the minimum living standard allowance and lived in extreme poverty, vulnerable children, at twice the minimum living allowance standard”. It can be inferred that welfare eligibility is poverty-based or income-based rather than a disability identity. As there is a strong correlation between poverty and disability, which has been confirmed by numerous studies, the beneficiaries of these policies are mostly PWDs. Some interviewees stated that they hardly received any benefits during the epidemic, mainly because they were not poor.

*I was fired during that time, and suddenly, there was no income, making my livelihood very difficult. I applied for social assistance from the community, hoping to obtain temporary assistance. However, my application was rejected because I am not poor, and my income level does not meet the conditions for being rescued. So, I hardly received any help or support. (C-5)*

## Conclusion and Discussion

We adopted qualitative research methods to explore the impact of war metaphors during the COVID-19 outbreak on PWDs in mainland China. Based on the analysis of interview data and policy documents, we found that the war metaphor dominated the principles and measures of epidemic prevention and control, which, in turn, affected the situation of PWDs. Although PWDs received formal or informal support during COVID-19, their situation worsened as they were further marginalized in the face of increasing social inequalities. Affected by the war metaphor, institutional segregation excludes PWDs from the epidemic prevention and control system. They are like outsiders, facing many visible and invisible social exclusions. Pragmatism derived from the war metaphor constructs the disabled as useless or non-contributory; therefore, PWDs are perceived as the minority who can be personally sacrificed. In a dominant “ableist” society, because of the lack of a disability lens and one-size-fits-all prevention and control measures, PWDs have not been given priority but neglected intentionally or unintentionally. In view of this, we need to reexamine the war metaphors during the COVID-19 pandemic and pay more attention to the voiceless and marginalized PWDs.

The war metaphor, as a rhetoric type that describes epidemic prevention and control with war discourse, aims to arouse the public's cognition of COVID-19 and create a sense of urgency and crisis, thus being regarded as an effective way of social mobilization. Undeniably, the war metaphor has played a key role in curbing the spread of this unprecedented public health crisis in China. However, it also has some negative and obscured effects. Although the war metaphor in medical discourse highlights the urgency and confrontational characteristics of war, it conceals its violent, compulsive, and destructive nature and ignores the irrationality and blind obedience it may cause (39). In addition, the one-sidedness of this overly exaggerated war metaphor has led to the concealment of social problems, such as the shutdown of economic production, local intensified social conflicts, and the suffering of vulnerable groups such as PWDs (40).

COVID-19 presents complex and multifaceted challenges for PWDs that require an inclusive and equitable response in the areas of public health and social responsibility. CRPD prohibits all discrimination, exclusion, or restriction based on disability and ensures that PWDs can enjoy free or affordable healthcare and services of the same quality and standard on an equal basis with others. Therefore, necessary laws and related policies need to be formulated to meet the basic life, social, emotional, and mental health needs of PWDs to ensure that they are not left behind in this crisis and are included (15). In addition, information and communication remain important weapons in the fight against COVID-19. Accordingly, CRPD requires appropriate facilities for PWDs to obtain information and meet their communication and information needs by providing them with specific technologies, languages, and accessible formats (29).

To improve the overall conditions of PWDs during COVID-19, rights-based strategies and community-based inclusive measures are key to ensuring that PWDs participate in and assume social responsibility in the process of policy design and implementation. It is necessary to give full play to the role of social organizations and support centers for PWDs to provide them with accessible and valuable information about COVID-19. Support needs to be provided for PWDs to use electronic information equipment so that they can strengthen their social interaction and connection with the outside world through online means as much as possible to reduce the sense of isolation. Further, for those profoundly disabled individuals who rely on family care, more attention should be paid not only to them but also to the family burden and the emotional health and quality of life of their family members. Correspondingly, a comprehensive social support system centered on families living with disabilities should be established.

This is a pioneering study on the impact of war metaphors on PWDs in the context of COVID-19. However, this study had two main limitations. First, it covered different types of PWDs and focused on exploring more general experiences that cannot go deep into individualized experiences about specific categories of PWDs. Subsequently, future studies can explore the unique experiences of this theme with a specific category of PWDs. Second, affected by COVID-19, face-to-face interviews cannot be conducted, and we have to recruit interviewees through online. However, in China, especially in the vast rural areas, affected by economic conditions and technical levels, many children and elderly persons with disabilities have limited ability to use related communication devices, which limits their use of WeChat, QQ, and Weibo. This makes it difficult for us to reach a wider range of people with disabilities through online recruitment. We adopted a qualitative method with a small-scale sample and whether the conclusions of this study are universally generalizable remains to be discussed. Therefore, we may not be able to overcome some of the limitations of qualitative research methods. Perhaps a hybrid research method combining qualitative and quantitative research can be applied to explore this important topic. Furthermore, when the COVID-19 is effectively prevented and controlled, the “legacy” and long-term effects of the war metaphor may still exist. Future study can carry out more in-depth exploration on this topic.

## Data Availability Statement

The raw data supporting the conclusions of this article will be made available by the authors, without undue reservation.

## Ethics Statement

The studies involving human participants were reviewed and approved by Academic Committee of Renmin University. Written informed consent to participate in this study was provided by the participants' legal guardian/next of kin.

## Author Contributions

R-XC: conceptualization, methodology, data collection and data analysis, writing-original draft, and writing-review and editing. Z-MG: conceptualization, methodology, data collection and data analysis, writing-original draft, writing-review and editing, and administration. S-LH: methodology, data collection and curation, and writing-original draft. W-ZT: methodology, data collection and curation, and funding acquisition. All authors contributed to the article and approved the submitted version.

## Funding

R-XC thanks the China Scholarship Council (CSC) for financial support (Grant 202006360233).

## Conflict of Interest

The authors declare that the research was conducted in the absence of any commercial or financial relationships that could be construed as a potential conflict of interest.

## Publisher's Note

All claims expressed in this article are solely those of the authors and do not necessarily represent those of their affiliated organizations, or those of the publisher, the editors and the reviewers. Any product that may be evaluated in this article, or claim that may be made by its manufacturer, is not guaranteed or endorsed by the publisher.
